# Comparison of Prognostic Impact between the Child-Pugh Score and Skeletal Muscle Mass for Patients with Liver Cirrhosis

**DOI:** 10.3390/nu9060595

**Published:** 2017-06-12

**Authors:** Hiroki Nishikawa, Hirayuki Enomoto, Akio Ishii, Yoshinori Iwata, Yuho Miyamoto, Noriko Ishii, Yukihisa Yuri, Kunihiro Hasegawa, Chikage Nakano, Takashi Nishimura, Kazunori Yoh, Nobuhiro Aizawa, Yoshiyuki Sakai, Naoto Ikeda, Tomoyuki Takashima, Ryo Takata, Hiroko Iijima, Shuhei Nishiguchi

**Affiliations:** Department of Hepatobiliary and Pancreatic disease, Department of Internal Medicine, Hyogo College of Medicine, 1-1, Mukogawacho, Nishinomiyashi, Hyogo 663-8501, Japan; nishikawa_6392@yahoo.co.jp (H.N.); akio0010@yahoo.co.jp (A.I.); yo-iwata@hyo-med.ac.jp (Y.I.); yuho.0818.1989@gmail.com (Y.M.); ishinori1985@yahoo.co.jp (N.I.); gyma27ijo04td@gmail.com (Y.Y.); hiro.red1230@gmail.com (K.H.); chikage@hyo-med.ac.jp (C.N.); tk-nishimura@hyo-med.ac.jp (T.N.); mm2wintwin@ybb.ne.jp (K.Y.); nobu23hiro@yahoo.co.jp (N.A.); sakai429@hyo-med.ac.jp (Y.S.); nikeneko@hyo-med.ac.jp (N.I.); tomo0204@yahoo.co.jp (T.T.); chano_chano_rt@yahoo.co.jp (R.T.); hiroko-i@hyo-med.ac.jp (H.I.); nishiguc@hyo-med.ac.jp (S.N.)

**Keywords:** liver cirrhosis, bioimpedance analysis, skeletal muscle mass index, Child-Pugh score, prognosis

## Abstract

Aims: To investigate the influence of skeletal muscle mass index (SMI) as determined by bioimpedance analysis (BIA) (appendicular skeletal muscle mass/(height)^2^) on survival by comparing the Child-Pugh score in patients with liver cirrhosis (LC, *n =* 383, average age = 65.2 years). Patients and methods: In terms of comparison of the effects of SMI and other markers on survival, we used time-dependent receiver operating characteristics (ROC) analysis. Results: The average SMI for male was 7.4 cm^2^/m^2^ whereas that for female was 6.0 cm^2^/m^2^ (*p* < 0.0001). As for the Child-Pugh score, five points were in the majority, both in males (51.7%, (106/205)) and females (44.9%, (80/178)). For both genders, the survival curve was well stratified according to SMI (*p* < 0.0001 for males and *p* = 0.0056 for females). In the multivariate analysis for survival, SMI and Child-Pugh scores were found to be significant both in males and females. In time-dependent ROC analyses, all area under the ROCs (AUROCs) for SMI in each time point were higher than those for Child-Pugh scores in males, while in females AUROCs for Child-Pugh scores at each time point were higher than those for SMI. Conclusion: SMI using BIA can be helpful for predicting outcomes, at least in male LC patients.

## 1. Introduction

The liver is the pivotal organ for metabolism and it metabolizes carbohydrates, lipids, and proteins, which are the so-called “three major nutrients” [1,2,3,4]. Liver cirrhosis (LC) is an end-stage form in liver diseases and LC is characterized by several metabolic or nutritional disorders and portal hypertension-related complications such as ascites or varices, all of which can lead to dismal clinical outcome [1,2,3,4]. Over the past two or three decades, numerous clinical and biochemical predictors have been proposed in an effort to more accurately predict the prognosis in LC patients and evaluate their short and long-term survival correctly [5,6,7,8,9]. The Child-Pugh scoring system and the Model for End-stage Liver Disease (MELD) scoring system are two major prognostic scoring systems in LC patients [5,6,7,8,9]. In particular, the MELD score is calculated by three easily available and reproducible laboratory tests and is useful for predicting outcomes for patients undergoing liver transplantation (LT) [5]. In our country, LT is not common due to the shortage of transplanted liver and the Child-Pugh scoring system has been preferably used for assessing prognosis in LC patients.

Sarcopenia is a clinical entity as determined by skeletal muscle mass loss and decline of muscle strength and this clinical entity has recently drawn much attention among clinicians owing to its significant deleterious impact on outcomes [10,11,12,13,14,15,16]. LC can be associated with secondary sarcopenia because of protein metabolic disorder and/or energy metabolic disorder [11,14,15]. Skeletal muscle mass loss can be linked to poorer clinical outcomes in LC patients, hepatocellular carcinoma (HCC) patients, or patients with other malignancies [17,18,19,20,21,22,23,24,25,26]. Skeletal muscle mass can be assessed by computed tomography (CT), magnetic resonance imaging (MRI), dual energy X-ray absorptiometry and bioimpedance analysis (BIA), which are consistent and accurate assessment modalities [14,15,17,19,27,28]. Among these modalities, BIA is particularly attractive since it can noninvasively determine body composition analysis in LC patients [14,15,17,19,27,28].

However, which of two prognostic markers (i.e., the Child-Pugh scoring system and skeletal muscle mass) has stronger influence on clinical outcomes in patients with LC remains unclear. Addressing these questions may be clinically of significance. The aim of this study was to investigate the influence of skeletal muscle mass as determined by data in BIA on survival compared with the Child-Pugh score in patients with LC.

## 2. Patients and Methods

### 2.1. Patients

The current study was a single center retrospective study. Between October 2005 and October 2015, a total of 529 LC individuals with BIA data available were admitted at the Division of Hepatobiliary and Pancreatic disease, Department of Internal Medicine, Hyogo College of Medicine, Hyogo, Japan. In our department, BIA (Inbody 720, Tokyo, Japan) was routinely performed in the resting and standing position principally on an outpatient basis for patients who consented to nutritional evaluation. In this analysis, skeletal muscle mass was evaluated using BIA data. Of these patients, patients with severe ascites (*n =* 24) were excluded from this study as body weight, body mass index (BMI), and skeletal muscle mass index (SMI) using BIA may be overestimated in these patients [29,30]. Twenty-three subjects had been lost to follow-up within one year after performing BIA and they were excluded from this analysis for avoiding bias. In the remaining 482 subjects, 99 had HCC on radiological findings at baseline and they were also excluded because presence of HCC can affect the interpretation of BIA data. A total of 383 subjects were therefore analyzed in the current study. Follow-up observation after BIA included periodical blood tests, radiological assessments by ultrasonography (US), CT, or MRI in order to detect HCC incidence every 3–6 months. There was no patient who underwent LT during observation period. LC was diagnosed using pathological findings, radiological findings such as US, CT, or MRI and/or laboratory data including liver fibrosis markers [31,32,33]. In patients with lower serum albumin level (less than 3.5 g/dL), liver supporting therapies including branched-chain amino acid (BCAA) treatment or late evening snack with BCAA enriched snacks were in consideration [4,34,35]. In patients with hepatitis virus-related LC, antiviral treatments including direct acting antivirals, interferon-based regimens or nucleoside analogues therapy were also in consideration [4,34]. SMI was calculated as reported elsewhere [28]. Briefly, SMI was defined as “appendicular skeletal muscle mass/(height (m))^2^” [28]. We retrospectively investigated the influence of SMI on survival in males and females, as compared with Child-Pugh scores, which was well established prognostic marker [7,8,9]. In terms of the comparison of the effects of SMI and other markers on survival, we used time-dependent receiver operating characteristics (ROC) analysis [36]. We also investigated parameters associated with overall survival (OS) in the univariate and multivariate analyses. HCC diagnosis and treatment choices for HCC were as reported elsewhere [37,38].

The ethical committee meeting in Hyogo College of Medicine acknowledged the current study protocol and this study strictly followed all regulations of the Declaration of Helsinki.

### 2.2. Statistical Analyses

Categorical parameters were compared by Fisher’s exact test. Continuous parameters were compared by unpaired *t*-test, Mann-Whitney *U* test, or Kruskal-Wallis test, as applicable. In continuous parameters, ROC curve analysis for survival was conducted for the purpose of setting the optimal cutoff point that is linked to maximal sum of specificity and sensitivity and we classified continuous parameters into two groups using these cutoff points, which was then treated as dichotomous covariates in the univariate analysis. Survival curve was created by using the Kaplan-Meier method and compared in the log-rank test. Parameters with *p* value < 0.05 in the univariate analysis were finally subjected to the multivariate analysis in the Cox proportional hazards model. OS was defined as the time interval from the date of performing BIA until death from any cause or the last follow-up visit. Additionally, we analyzed time-dependent ROC curves of SMI, Child-Pugh scores, and variables which revealed to be significant in the multivariate analysis for survival, and compared between area under the ROCs (AUROCs) for above parameters in each time point (two-, three-, four-, five-, six-, and seven-years) [36].

Data are shown as the average ± standard deviation (SD) unless otherwise mentioned. Statistical significance was set at *p* < 0.05. Statistical analysis was performed with the JMP 11 (SAS Institute Inc., Cary, NC, USA).

## 3. Results

### 3.1. Baseline Characteristics

**Figure 1:**
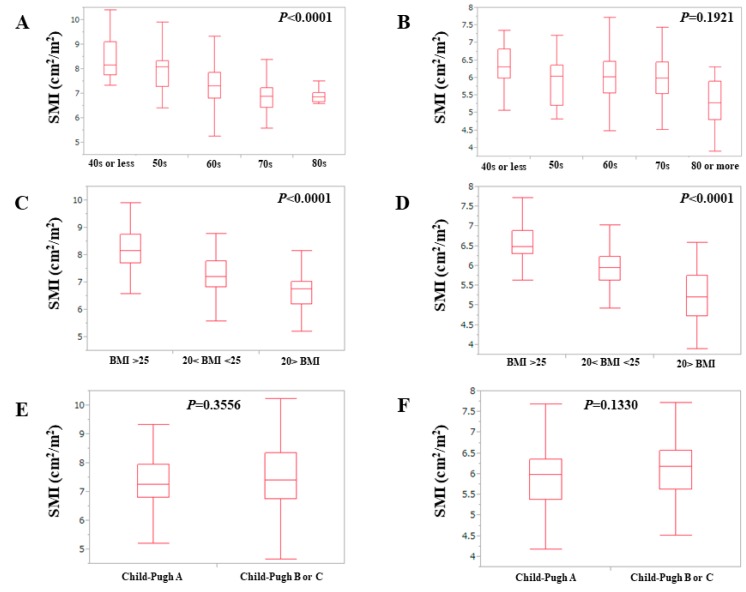
(**A**,**B**) Relationship between SMI and age in males (**A**) and females (**B**). (**C**,**D**) Relationship between SMI and BMI in males (**C**) and females (**D**). (**E**,**F**) Relationship between SMI and Child-Pugh classification in males (**E**) and females (**F**). SMI: skeletal muscle mass index; BMI: body mass index.

The baseline characteristics of the analyzed subjects (*n =* 383) are presented in Table 1. They included 205 males and 178 females with an average ± SD age of 65.2 ± 10.3 years. The median follow-up periods were 3.2 years (range: 0.2–10.7 years). The average ± SD value in SMI for male was 7.4 ± 0.9 cm^2^/m^2^ whereas that for female was 6.0 ± 0.7 cm^2^/m^2^ (*p <* 0.0001). According to the Asian Working Group for Sarcopenia criteria (AWGS), the cut-off values for SMI are 7.0 kg/m^2^ for male and 5.7 kg/m^2^ for female. [28] The proportion of decreased SMI (D-SMI: less than each cutoff value as defined by AWGS criteria) in male was 36.1% (74/205) and that in female was 34.8% (62/178). A total of 136 patients (35.5%) had D-SMI. As for Child-Pugh scores, five points was in the majority, both in males (51.7%, (106/205)) and females (44.9%, (80/178)). In males, SMI significantly correlated with age (overall significance, *p* < 0.0001), while in females it did not (overall significance, *p =* 0.1921) (Figure 1A,B). In both males and females, SMI significantly correlated with BMI (*p* values, both <0.0001) (Figure 1C,D). In both males (*p =* 0.3716) and females (*p =* 0.1330), SMI did not significantly correlate with the Child-Pugh classification (Figure 1E,F).

### 3.2. Cumulative OS Rates for the Entire Cohort, Male and Female According to SMI

**Figure 2:**
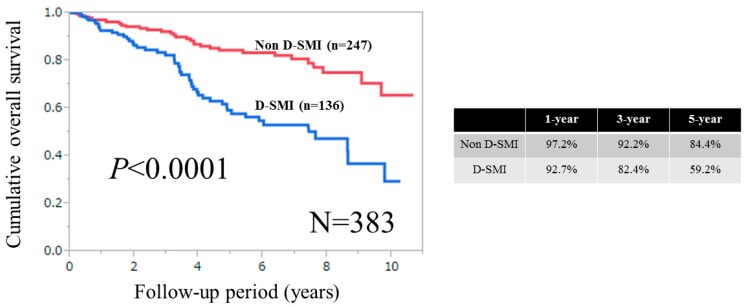
Cumulative overall survival for the entire cohort (*n =* 383). The one-, three-, and five-year cumulative OS rates were 92.7%, 82.4% and 59.2%, respectively, in patients with decreased SMI (D-SMI), and 97.2%, 92.2% and 84.4%, respectively, in patients without D-SMI (*p <* 0.0001). D-SMI was defined according to the Asian Working Group for Sarcopenia criteria. The cut-off values for SMI are 7.0 kg/m^2^ for males and 5.7 kg/m^2^ for females. SMI: skeletal muscle mass index; D-SMI: decreased SMI.

**Figure 3:**
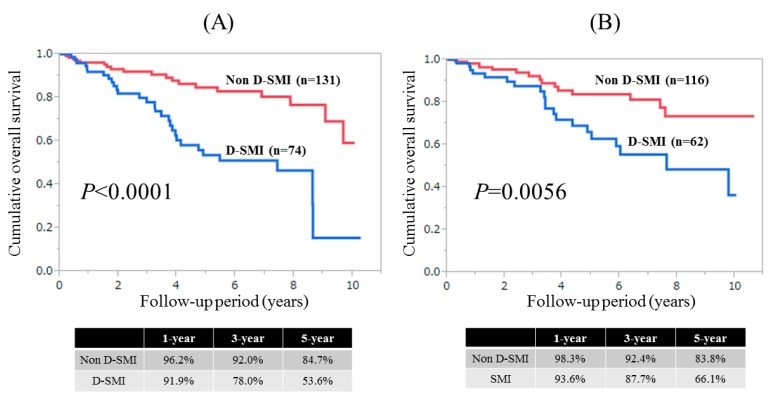
Cumulative overall survival for males (**A**) *n =* 205 and for females (**B**) *n =* 178 in patients with and without D-SMI. SMI: skeletal muscle mass index; D-SMI: decreased SMI.

For the entire cohort (*n =* 383), the one-, three-, and five-year cumulative OS rates were 92.7%, 82.4%, and 59.2%, respectively, in patients with D-SMI, and 97.2%, 92.2%, and 84.4%, respectively, in patients without D-SMI (*p <* 0.0001) (Figure 2). For males (*n =* 205), the one-, three-, and five-year cumulative OS rates were 91.9%, 78.0%, and 53.6%, respectively, in patients with D-SMI, and 96.2%, 92.0%, and 84.7%, respectively, in patients without D-SMI (*p <* 0.0001) (Figure 3A). For females (*n =* 178), the one-, three-, and five-year cumulative OS rates were 93.6%, 87.7%, and 66.1%, respectively, in patients with D-SMI, and 98.3%, 92.4%, and 83.8%, respectively, in patients without D-SMI (*p =* 0.0056) (Figure 3B).

Using ROC analysis for survival, the optimal cut-off point for SMI was 7.0 cm^2^/m^2^ in males (AUROC = 0.672, sensitivity = 64.6%, specificity = 69.4%) and 5.4 cm^2^/m^2^ in females (AUROC = 0.658, sensitivity = 45.7%, specificity = 83.9%). When the cut-off value of 5.4 cm^2^/m^2^ was adapted to our female patients (cut-off value for female in WAGS; 5.7 cm^2^/m^2^), similar results were obtained. That is, patients with D-SMI had significantly poorer survival rates than those without D-SMI (*p =* 0.0076).

### 3.3. Causes for Death for Males and Females

In male, during the observation period, 48 patients (23.4%) died. The causes for death were liver failure in 28 patients, HCC progression in 10 patients and miscellaneous causes in 10 patients. In female, during the observation period, 35 patients (19.7%) died. The causes for death were liver failure in 28 patients, HCC progression in four patients and miscellaneous causes in three patients.

### 3.4. Univariate and Multivariate Analyses of Parameters Contributing to OS for Males

Univariate analysis identified the following factors as significantly associated with OS for males: age (*p =* 0.0041); SMI (*p <* 0.0001); Child-Pugh score (*p =* 0.0252); aspartate aminotransferase (*p =* 0.0150); alanine aminotransferase (ALT) (*p =* 0.0407); serum albumin (*p =* 0.0011); serum sodium (*p =* 0.0133); serum creatinine (*p =* 0.0088); and BMI (*p =* 0.0041) (Table 2a). Since the Child-Pugh score includes serum albumin, it was not entered into the multivariate analysis, and since age and BMI significantly correlated with SMI, they were also excluded in the multivariate analysis to avoid the effect of collinearity. The hazard ratios (HRs) and 95% confidence intervals (CIs) calculated by using multivariate analysis for the six significant variables (*p <* 0.05) in the univariate analysis are presented in Table 2b. SMI (*p =* 0.0005) and Child-Pugh score (*p =* 0.0424) were found to be significant predictors related to OS in the multivariate analysis (Table 2b).

### 3.5. Univariate and Multivariate Analyses of Parameters Contributing to OS for Females

Univariate analysis identified the following parameters as significantly associated with OS for females: age (*p =* 0.0214); SMI (*p =* 0.0076); Child-Pugh score (*p =* 0.0009); ALT (*p =* 0.0104); serum albumin (*p =* 0.0003); prothrombin time (PT) (*p =* 0.0355); platelet count (*p =* 0.0333); triglyceride (*p =* 0.0011); serum creatinine (*p =* 0.0137); BMI (*p =* 0.0270) and presence of ascites (*p <* 0.0001) (Table 3a). As the Child-Pugh score includes serum albumin, PT, and ascites, they were not entered into the multivariate analysis, and because BMI significantly correlated with SMI, it was also excluded in the multivariate analysis to avoid the effect of collinearity. The HRs and 95% CIs calculated by using multivariate analysis for the seven significant variables (*p <* 0.05) in the univariate analysis are presented in Table 3b. SMI (*p =* 0.0016) and Child-Pugh scores (*p <* 0.0001) were found to be significant predictors related to OS in the multivariate analysis (Table 3b).

### 3.6. Time-Dependent ROC Analyses for OS in Males

**Figure 4:**
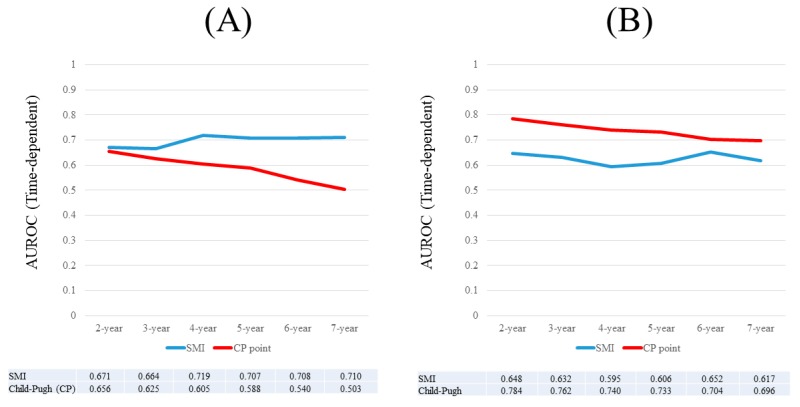
(**A**) Results for time-dependent ROC analyses at two-, three, four-, five-, six-, and seven-years of SMI and Child-Pugh scores for males; (**B**) Results for time-dependent ROC analyses at two-, three-, four-, five-, six-, and seven-years of SMI and Child-Pugh scores for females. ROC; receiver operating characteristics, AUROC; area under the ROC, SMI; skeletal muscle mass index, CP; Child-Pugh.

Results for time-dependent ROC analyses at two-, three-, four-, five-, six-, and seven-years of SMI and the Child-Pugh scores for males are shown in Figure 4A. All AUROCs for SMI at each time point were higher than those for Child-Pugh scores, denoting that SMI had consistently superior predictive ability for OS over Child-Pugh scores.

### 3.7. Time-Dependent ROC Analyses for OS in Females

Results for time-dependent ROC analyses at two-, three-, four-, five-, six-, and seven-years of SMI and Child-Pugh score for female were shown in Figure 4B. All AUROCs for Child-Pugh scores at each time point were higher than those for SMI, denoting that Child-Pugh scores had consistently superior predictive ability for OS over SMI.

### 3.8. Time-Dependent ROC Analyses for OS in Male Patients with Child-Pugh A

**Figure 5:**
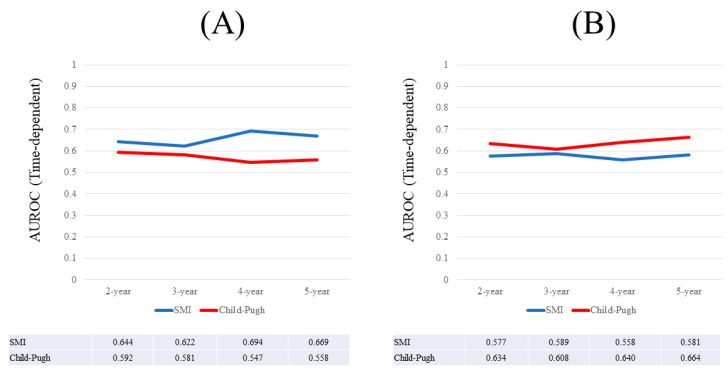
(**A**) Results for time-dependent ROC analyses at two-, three-, four-, and five-years of SMI and Child-Pugh scores for male Child-Pugh A patients; (**B**) Results for time-dependent ROC analyses at two-, three-, four-, and five-years of SMI and Child-Pugh scores for female Child-Pugh A patients. ROC; receiver operating characteristics, AUROC; area under the ROC, SMI; skeletal muscle mass index.

Results for time-dependent ROC analyses at two-, three-, four-, and five-years of SMI and Child-Pugh scores for male Child-Pugh A patients (*n =* 148) are shown in Figure 5A. All AUROCs for SMI in each time point were higher than those for Child-Pugh scores, denoting that SMI had consistently superior predictive ability for OS over Child-Pugh scores.

### 3.9. Time-Dependent ROC Analyses for OS in Female Patients with Child-Pugh A

Results for time-dependent ROC analyses at two-, three-, four-, and five-years of SMI and Child-Pugh scores for female Child-Pugh A patients (*n =* 131) are shown in Figure 5B. All AUROCs for Child-Pugh score in each time point were higher than those for SMI, denoting that Child-Pugh scores had consistently superior predictive ability for OS over SMI.

### 3.10. Time-Dependent ROC Analyses for OS in Male Patients with Child-Pugh B or C

**Figure 6:**
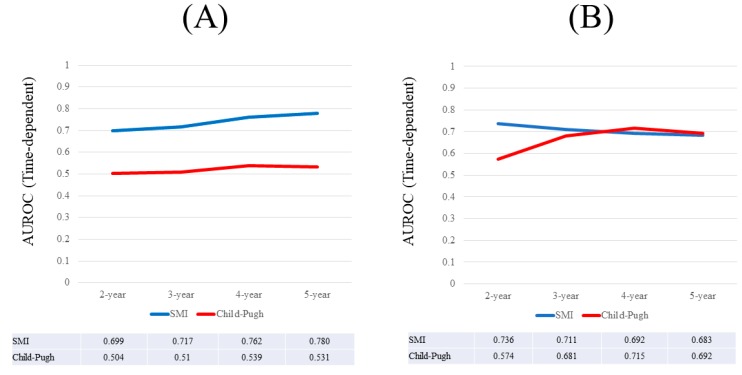
(**A**) Results for time-dependent ROC analyses at two-, three-, four-, and five-years of SMI and Child-Pugh score for male Child-Pugh B or C patients; (**B**) Results for time-dependent ROC analyses at two-, three-, four-, and five-years of SMI and Child-Pugh scores for female Child-Pugh B or C patients. ROC; receiver operating characteristics, AUROC; area under the ROC, SMI; skeletal muscle mass index.

Results for time-dependent ROC analyses at two-, three-, four-, and five-years of SMI and Child-Pugh scores for male Child-Pugh B or C patients (*n =* 57) are shown in Figure 6A. All AUROCs for SMI at each time point were higher than those for Child-Pugh scores, denoting that SMI had consistently superior predictive ability for OS over Child-Pugh scores.

### 3.11. Time-Dependent ROC Analyses for OS in Female Patients with Child-Pugh B or C

Results for time-dependent ROC analyses at two-, three-, four-, and five-years of SMI and Child-Pugh scores for female Child-Pugh B or C patients (*n =* 47) are shown in Figure 6B. At four- and five-years, Child-Pugh scores had higher AUROC than SMI.

## 4. Discussion

To the best of our knowledge, this is the first comparative study in SMI and Child-Pugh scores on clinical outcomes in LC patients. The Child-Pugh scoring system is a well-established prognostic system in LC patients [5,6,7,8,9]. While, SMI in LC has been currently attracting much attention owing to its well predictive performance [14,15,16,17,19,27,28]. Investigation into the influence of SMI on outcomes in LC patients is pivotal and, particularly, clarifying which of these two markers has stronger predictive impact for LC is clinically essential in light of creating a novel prognostic system in LC patients. We, therefore, conducted this comparative analysis to address this question. Since skeletal muscle mass significantly can differ between male and female, we analyzed and discussed separately in males and females.

In our results, for males, SMI and Child-Pugh scores were revealed to be significant for OS in the multivariate analysis, and all AUROCs for SMI at each time point were higher than those for Child-Pugh scores. Additionally, in subgroup analyses for male patients with Child-Pugh A or Child-Pugh B or C, SMI had consistently higher AUROCs than the Child-Pugh score. For females, similar results were obtained in the multivariate analysis, however, all AUROCs for Child-Pugh scores at each time point were higher than those for SMI. These results denote that SMI as a predictor can perform well as compared with Child-Pugh score at least in male LC patients. Our current results may provide new information and shed some lights for the better comprehension of prognosis in LC patients. The significant difference in baseline characteristics in such as SMI, age, serum sodium, and serum creatinine between males and females can explain the different results according to gender. In particular, aging can be a significant predictor in LC patients [14,39,40]. Our country is an aging country [39,40]. The reasons for results in time-dependent ROC analysis in females may be attributed to our results that SMI did not significantly correlate with age in females.

In view of our current results, some interventional therapies, including BCAA therapy, testosterone therapy, or exercise, can be considered especially in male LC patients with lower skeletal muscle mass for ameliorating prognosis [13,35,41,42,43]. However, firm recommendations for LC patients are not currently available. As for cut-off value for BIA, AWGS recommends 2SDs below the average muscle mass of young adults (7.0 cm^2^/m^2^ for male and 5.7 cm^2^/m^2^ for female) [28]. In our outcome based ROC analysis, the optimal cut-off points for survival were 7.0 cm^2^/m^2^ for males and 5.4 cm^2^/m^2^ for females, which were quite similar to recommendations in AWGS and their recommendations were well validated in our analysis. From the viewpoint of outcome-based analysis, our results may be worthy of reporting.

As described above, it is of note that SMI significantly correlated with age in males, but not in females. In general, changes in muscle mass occur with aging and muscle mass loss is a common condition which is recognized as a part of aging [44]. However, our results showed that the rates of muscle mass decline can vary according to gender, suggesting that factors other than aging, such as diet intake and lifestyle, may influence the maintenance of healthy muscle mass [45]. On the other hand, although the liver transplantation allocation system utilizes MELD score to prioritize organs to the most ill subjects, MELD scores do not perform better than Child-Pugh scores in non-transplant settings. In other words, MELD scores can perform well in decompensated LC rather than compensated LC [8]. Due to the high proportion of patients with Child-Pugh A in our cohort, we did not include MELD scores in the analysis.

Several limitations must be acknowledged in our current analysis. First, this study is a single-center retrospective observational study utilizing data for BIA and parameters reflecting muscle function such as hang grip strength or walking speed were not assessed in this analysis. In future studies, both skeletal muscle mass and muscle function should be evaluated in outcome based analyses. Second, patients with severe ascites were excluded from our analysis because SMI can be overestimated in these patients. Thus, the number of Child-Pugh C patients was rather small and our results cannot be adapted to such patients. Body composition analyses using BIA can be challenging in LC patients with severe ascites. Finally, various treatments for underlying liver diseases were performed during follow-up period in each patient, potentially creating bias. However, our study results denoted that SMI had higher predictive ability, at least in male LC patients. Results in time-dependent ROC analysis support our assertion for the predictive superiority of SMI over Child-Pugh scores in males.

In conclusion, SMI can be accessible for predicting outcomes, at least in male LC patients. Some interventions for male patients with lower SMI may be recommended.

## Figures and Tables

**Table 1:** Baseline characteristics (*n =* 383).

Variables	Number or Average ± SD	Male (*n =* 205)	Female (*n =* 178)	*p* Value (Male vs. Female)
Age (years)	65.2 ± 10.3	64.1 ± 10.8	66.4 ± 9.5	0.0403
Body mass index (kg/m^2^)	23.3 ± 3.9	23.4 ± 3.8	23.2 ± 4.0	0.6028
Skeletal muscle mass index (cm^2^/m^2^)	6.7 ± 1.1	7.4 ± 0.9	6.0 ± 0.7	<0.0001
Causes of liver disease	32/235/116	22/122/61	10/113/55	0.2043
Hepatitis B/Hepatitis C/others				
Child-Pugh scores, 5/6/7/8/9/10/11	186/93/58/28/13/3/2	106/42/31/15/6/3/2	80/51/27/13/7/0/0	0.2587
Total bilirubin (mg/dL)	1.3 ± 1.1	1.3 ± 1.3	1.2 ± 0.7	0.2319
Serum albumin (g/dL)	3.7 ± 0.53	3.7 ± 0.54	3.6 ± 0.51	0.3897
Prothrombin time (%)	77.0 ± 13.8	77.5 ± 13.4	76.4 ± 14.2	0.4532
Platelets (×10^4^/mm^3^)	10.6 ± 5.5	10.5 ± 5.3	10.7 ± 5.7	0.8093
Serum sodium (mmol/L)	139.7 ± 2.5	139.3 ± 2.5	140.2 ± 2.6	0.0004
Serum creatinine (mg/dL)	0.74 ± 0.51	0.84 ± 0.65	0.62 ± 0.22	<0.0001
Total cholesterol (mg/dL)	154.0 ± 36.8	152.8 ± 35.4	155.2 ± 38.4	0.5250
Triglyceride (mg/dL)	90.5 ± 45.1	95.5 ± 53.0	84.8 ± 33.0	0.2144
AST (IU/L)	49.3 ± 34.8	49.4 ± 32.3	49.2 ± 37.5	0.9417
ALT (IU/L)	42.6 ± 38.9	43.9 ± 34.2	41.0 ± 43.7	0.0951
Fasting blood glucose (mg/dL)	110.9 ± 34.7	114.8 ± 39.9	106.4 ± 27.0	0.0101
Ascites, yes/no	44/339	25/180	19/159	0.7484

Data are expressed as number or average ± standard deviation (SD). AST: aspartate aminotransferase; ALT: alanine aminotransferase.

**Table 2:** (**a**) Univariate analyses of factors linked to overall survival for males (*n =* 205). (**b**) Multivariate analyses of factors linked to overall survival for male.

**(a)**			
**Variables**	**Number of Each Category**		**Univariate**
			***p* Value**
Age (years) ≥ 70, yes/no	91/114		0.0041
Cause of liver diseases, B/C/others	22/122/61		0.2746
SMI ≥ 7.0 cm^2^/m^2^, yes/no	127/78		<0.0001
Child-Pugh score ≥ 6, yes/no	99/106		0.0252
AST ≥ 29 IU/L, yes/no	146/59		0.0150
ALT ≥ 47 IU/L, yes/no	63/142		0.0407
Serum albumin ≥ 3.7 g/dL, yes/no	113/92		0.0011
Total bilirubin ≥ 2.0 mg/dL, yes/no	29/176		0.1539
Prothrombin time ≥ 77.1%, yes/no	111/94		0.4521
Platelet count ≥ 9.4 × 10^4^/mm^3^, yes/no	101/104		0.2360
Total cholesterol ≥ 124 mg/dL, yes/no	165/40		0.2398
Triglyceride ≥ 56 mg/dL, yes/no	170/35		0.0649
Serum sodium ≥ 138 mmol/L, yes/no	166/39		0.0133
Fasting blood glucose ≥ 97 mg/dL, yes/no	145/60		0.4942
Serum creatinine ≥ 0.78 mg/dL, yes/no	84/121		0.0088
Body mass index ≥ 23.4 kg/m^2^, yes/no	91/114		0.0041
Ascites, yes/no	25/180		0.0712
**(b)**			
**Variables**	**Multivariate Analysis**		
	**Hazard Ratio**	**95% CI**	***p* Value**
SMI (per one cm^2^/m^2^)	0.571	0.416–0.777	0.0005
AST (per one IU/L)	1.005	0.987–1.022	0.5683
ALT (per one IU/L)	0.990	0.972–1.008	0.3002
Child-Pugh score (per one point)	1.270	1.020–1.520	0.0424
Serum sodium (per one mmol/L)	0.912	0.807–1.033	0.1481
Serum creatinine (per one mg/dL)	1.104	0.769–1.397	0.4914

CI: confidence interval; SMI: skeletal muscle mass index; AST: aspartate aminotransferase; ALT: alanine aminotransferase.

**Table 3:** (**a**) Univariate analyses of factors linked to overall survival for female (*n =* 178). (**b**) Multivariate analyses of factors linked to overall survival for female.

**(a)**			
**Variables**	**Number of Each Category**		**Univariate**
			***p* value**
Age (years) ≥ 77, yes/no	22/156		0.0214
Cause of liver diseases, B/C/others	10/113/55		0.5037
SMI ≥ 5.4 cm^2^/m^2^, yes/no	139/39		0.0076
Child-Pugh score ≥ 6, yes/no	98/80		0.0009
AST ≥ 80 IU/L, yes/no	20/158		0.0544
ALT ≥ 58 IU/L, yes/no	32/146		0.0104
Serum albumin ≥ 3.4 g/dL, yes/no	117/61		0.0003
Total bilirubin ≥ 2.3 mg/dL, yes/no	10/168		0.7607
Prothrombin time ≥ 73.7%, yes/no	103/75		0.0355
Platelet count ≥ 9.7 ×10^4^/mm^3^, yes/no	92/86		0.0333
Total cholesterol ≥ 176 mg/dL, yes/no	49/129		0.0558
Triglyceride ≥ 72 mg/dL, yes/no	108/70		0.0011
Serum sodium ≥ 139 mmol/L, yes/no	143/35		0.4064
Fasting blood glucose ≥ 89 mg/dL, yes/no	142/36		0.2428
Serum creatinine ≥ 0.63 mg/dL, yes/no	66/112		0.0137
Body mass index ≥ 23.4 kg/m^2^, yes/no	78/100		0.0270
Ascites, yes/no	19/159		<0.0001
**(b)**			
**Variables**	**Multivariate Analysis**		
	**Hazard ratio**	**95% CI**	***p* Value**
Age (per one year)	1.018	0.977–1.063	0.3998
SMI (per one cm^2^/m^2^)	0.450	0.270–0.731	0.0016
ALT (per one IU/L)	0.987	0.967–1.107	0.0506
Platelet count (per one × 10^4^/mm^3^)	0.919	0.828–1.008	0.0943
Child-Pugh score (per one point)	1.938	1.400–2.690	<0.0001
Triglyceride (per one mg/dL)	0.998	0.983–1.011	0.7760
Serum creatinine (per one mg/dL)	2.546	0.413–10.860	0.2545

CI: confidence interval; SMI: skeletal muscle mass index; AST: aspartate aminotransferase; ALT: alanine aminotransferase.

## Abbreviations

LC: liver cirrhosis; MELD: Model for End-stage Liver Disease; LT: liver transplantation; HCC: hepatocellular carcinoma; CT: computed tomography; MRI: magnetic resonance imaging; BIA: bioimpedance analysis; SMI: skeletal muscle mass index; BMI: body mass index; US: ultrasonography; BCAA: branched-chain amino acid; ROC: receiver operating characteristic curve; OS: overall survival; AUROC: area under the receiver operating characteristic curve; SD: standard deviation; AWGS: Asian Working Group for Sarcopenia criteria; D-SMI: decreased SMI; ALT: alanine aminotransferase; HR: hazard ratios; CI: confidence interval.
